# The Discrepancy Between Standard Histologic WHO Grading of Meningioma and Molecular Profile: A Single Institution Series

**DOI:** 10.3389/fonc.2022.846232

**Published:** 2022-03-01

**Authors:** Amanda M. Roehrkasse, Jo Elle G. Peterson, Kar-Ming Fung, Panayiotis E. Pelargos, Ian F. Dunn

**Affiliations:** ^1^ Dunn Laboratory, Department of Neurosurgery, University of Oklahoma Health Sciences Center, Oklahoma City, OK, United States; ^2^ Department of Pathology, University of Oklahoma Health Sciences Center, Oklahoma City, OK, United States; ^3^ Stephenson Cancer Center, University of Oklahoma Health Sciences Center, Oklahoma City, OK, United States

**Keywords:** meningioma, *TERT*, *BAP1*, *CDKN2A/B*, microarray, genetic profiling, copy number alteration, CNS tumors

## Abstract

**Introduction:**

Meningiomas are the most common primary central nervous system (CNS) tumor. They are most often benign, but a subset of these can behave aggressively. Current World Health Organization (WHO) guidelines classify meningiomas into three grades based on the histologic findings and presence or absence of brain invasion. These grades are intended to guide treatment, but meningiomas can behave inconsistently with regard to their assigned histopathological grade, influencing patient expectations and management. Advanced molecular profiling of meningiomas has led to the proposal of alternative molecular grading schemes that have shown superior predictive power. These include methylation patterns, copy number alterations, and mutually exclusive driver mutations affecting oncogenes, including *BAP1, CDKN2A/B*, and the *TERT* promoter, which are associated with particularly aggressive tumor biology. Despite the evident clinical value, advanced molecular profiling methods are not widely incorporated in routine clinical practice for meningiomas.

**Objective:**

To assess the degree of concordance between the molecular profile of meningiomas and the histopathologic WHO classification, the current method of predicting meningioma behavior.

**Methods:**

In a two-year single-institution experience, we used commercially available resources to determine molecular profiles of all resected meningiomas. Copy number aberrations and oncogenic driver mutations were identified and compared with the histopathologic grade.

**Results:**

One hundred fifty-one total meningioma cases were included for analysis (85.4% WHO grade 1, 13.3% WHO grade 2, and 1.3% grade 3). Chromosomal analysis of 124 of these samples showed that 29% of WHO grade 1 tumor featured copy number profiles consistent with higher grade meningioma, and 25% of WHO grade 2 meningiomas had copy number profiles consistent with less aggressive tumors. Furthermore, 8% harbored mutations in *TERT, CDKN2A/B*, or *BAP1* of which 6% occurred in grade 1 meningiomas.

**Conclusions:**

Routine advanced molecular profiling of all resected meningiomas using commercially available resources allowed for identification of a significant number of meningiomas whose molecular profiles were inconsistent with WHO grade. Our work shows the clinical value of integrating routine molecular profiling with histopathologic grading to guide clinical decision making.

## Introduction

Meningiomas, named for their cell of origin, are the most common intracranial central nervous system (CNS) tumors in adults representing approximately one-third of all primary adult CNS tumors (1–3). The World Health Organization (WHO) Classification of Tumours of the CNS subdivides meningiomas into three grades (i.e., 1, 2, or 3) that are intended to correlate with prognosis and guide management (4, 5). The majority of meningiomas fall into 9 histologic subtypes comprising grade 1 meningiomas (see **Supplementary Table 1**), which have a reported progression-free survival (PFS) of 75%-90%. Grade 2 meningiomas are defined by either pathognomonic histology (i.e., clear cell or chordoid), or a grade 1 histologic subtype with a mitotic rate of 4-19 mitoses per 10 microscopic high-powered fields (HPF), brain invasion, and/or the presence of three of five atypical features as defined by the WHO. Grade 2 meningiomas have higher rates of recurrence and morbidity with a PFS of 23-78%. Grade 3 meningiomas are considered malignant with a PFS of 0% and are defined as a grade 1 histologic subtype with a mitotic index of ≥20 per 10 HPF, or the presence of rhabdoid or papillary histologic subtypes (4–6).

In current practice, WHO grade and extent of resection are the most widely used metrics to predict tumor behavior and guide management of meningiomas (6, 7). Most management paradigms involve adjuvant radiation after total or subtotal resection of grade 3 meningiomas. The use of radiation therapy as an adjunct treatment for recurrent grade 1 as well as grade 2 meningiomas following gross-total or sub-total resection is variable but commonly deployed, largely based on institutional retrospective series (7–9). An emerging observation is that meningiomas can behave inconsistently with the assigned WHO grade: grade 1 tumors can recur or behave aggressively despite successful gross total resection, while tumors with more advanced grades may have favorable natural histories (10, 11).

A developing understanding of the molecular landscape of meningioma suggests that WHO grade alone may not provide an adequate prediction of tumor behavior, for surveillance or adjuvant treatment planning considerations. Several recent publications have proposed alternative grading systems that incorporate genomic and/or epigenomic data in order to better predict meningioma behavior, particularly targeting those that do not behave in concordance with their assigned WHO grade (10–14). These grading systems incorporate molecular data including sequence alterations, methylation data and copy number alterations, among other modalities, and have shown a compelling ability to predict meningioma recurrence and progression-free survival when compared with the WHO histologic-based schema alone.

While methylation data are highly predictive, their availability is limited compared to genomic methods that are currently available to most centers. In particular, emerging data on mutational profiling and copy number variation have identified specific molecular features that, when present, are correlated with higher recurrence risks and poorer prognoses. The evolving molecular profiling of meningiomas seeks to minimize interobserver variability inherent to applying subjective phenotypic grading criteria and creates smaller, more homogenous subclassifications (15, 16). Several recurring oncogenic mutations have been identified that are relatively rare but associated with particularly aggressive behavior, even when encountered in histologically low-grade meningiomas. These include alterations in the *BAP1*, *CDKN2A/B*, and mutations within the promotor region of the *TERT* genes (17–20). Copy number alterations have also been shown to have strong predictive potential, with their ease of acquisition at most centers (either in-house or *via* vendor) enhancing their appeal (10, 13). These studies have highlighted that, regardless of histology, a greater degree of chromosomal disruption is more reliable in the forecasting of recurrence and outcome than WHO grading alone.

The rapid advances in molecular profiling of meningiomas and evident clinical benefit of acquiring genetic data for clinical management has led to incorporation of molecular designations in the most recent update to the WHO classification (21). In light of the putative superiority of molecular profiling in predicting meningioma behavior, we sought to determine how routine incorporation of readily obtainable advanced molecular profiling (copy number and mutational data) would compare with assigned WHO histopathologic grade in a prospective series of meningiomas.

## Materials and Methods

### Human Subjects

All methods were approved by the institutional review board (IRB) of the University of Oklahoma Health Sciences Center (OUHSC) (IRB protocol number 10195). All patients who underwent their index surgery with a histopathologic diagnosis of meningioma following CNS tumor resection from May 2019-April 2021 were included in the study. Patient data including demographics, tumor location, and history of prior resection were collected by retrospective chart review.

### Histopathologic Grading

Following routine pathology processing, resected meningiomas were assigned a histopathologic grade by board-certified neuropathologists within the Department of Pathology at OU Health according to the guidelines set forth in the revised 4^th^ edition of the WHO Classification of Tumours of the CNS published in 2016 (4, 5). Immunohistochemical stains, such as glial fibrillary acid protein (GFAP) and epithelial membrane antigen (EMA), were performed on a case-by-case basis as deemed necessary for diagnostic evaluation. Ki-67 immunostaining was performed on at least one block in all cases. All samples were analyzed, graded, and independently confirmed by two neuropathologists (JGP and KMF).

### Molecular Profiling by Next-Generation Sequencing and Chromosomal Microarray

Beginning in November 2019, chromosomal profiling and mutational data were prospectively acquired on all meningiomas in collaboration with the Mayo Clinic Laboratories, where chromosomal profiling and sequencing of 118 CNS tumor-associated genes was performed. Based on prior studies that investigated clinical progression of genetic subgroups of meningiomas, grade 1 meningiomas that harbored mutations in *BAP1*, *CDKN2A* or *B*, or the *TERT* promoter were considered to have molecular profiles that were higher risk and inconsistent with histopathologic grading (17, 19, 20, 22). Copy number data consistent with a higher grade (2/3) meningioma was defined as any loss of chromosome 1p, 3p, 4p/q, 6p/q, 10p/q, 14q, 18p/q, and/or 19p/q. Normal copy number and minor copy number alterations not involving the chromosomes listed, monosomy of chromosome 22, and cases with multiple polysomies consistent with angiomatous meningioma were considered to be consistent with grade 1 meningiomas. (6, 10, 11, 23).

### Tumor Location

To assign tumor location, all available imaging was reviewed. Meningiomas were classified based on their location into anterior skull base, middle skull base – medial, middle skull base – lateral, anteromedial posterior cranial fossa, posterolateral posterior cranial fossa, spinal, anterior convexity (anterior to central sulcus), posterior convexity (posterior to central sulcus), falcine/parasagittal, tentorial, tentorial sinus, peritorcular, intraventricular, or multifocal. Meningiomas were further classified based on their sublocation (i.e., anterior clinoid process, posterior clinoid process, clival, etc.) (**Supplementary Figure 1**).

### Data Analysis

Descriptive statistics, scatter plots, and figures were generated using GraphPad Prism (GraphPad Software, San Diego, CA). Summary statistics are reported as counts or proportions for categorical variables. For continuous variables (e.g., Ki-67) mean and standard deviation are shown in figures. Statistical analysis of copy number alterations and Ki-67 between meningiomas with high-grade and low-grade copy number variations were done by one-way analysis of variance (ANOVA) with Tukey’s *post-hoc* test for multiple comparisons. Significance was defined as a p-value of less than 0.05.

## Results

### A Two-Year Cohort of Resected Meningiomas Used to Assess the Clinical Value of Routine Advanced Molecular Profiling

**Figure 1:**
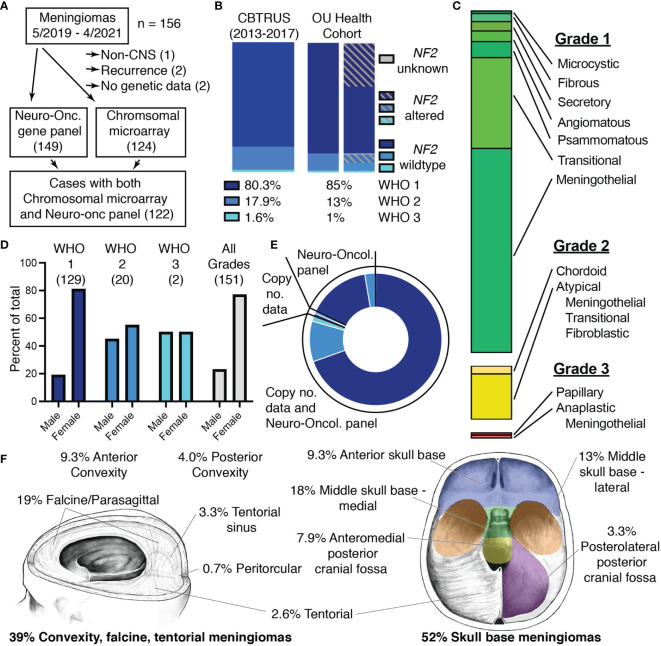
Characteristics of the two-year OU Health meningioma cohort. **(A)** Flowchart depicting selection of meningiomas for the OU Health cohort and molecular data collected with the number of cases shown in parentheses. **(B)** Percentages and *NF2*-status of meningiomas assigned to each WHO grade in the meningioma cohort with national data from the 2020 CBTRUS statistical report shown for reference (2). **(C)** Histologic architecture seen in the meningioma cohort. Proportions of Grade I (green), Grade II (yellow), and Grade III (red) meningiomas are shown. **(D)** Proportion of male and female patients for each WHO Grade and the total cohort. The number of patients in each category is shown in parentheses. **(E)** Plot depicting the proportion of cases with data from either the Neuro-Oncology Panel, copy number data (chromosomal microarray), or cases with Neuro-Oncology Panel as well as copy number data. Shades of blue correspond to WHO Grade I-III as in panel **(B)**. **(F)** Location of meningiomas included in the cohort. Meningiomas classified as spinal (2.6%), optic nerve (0.7%), and cases with multiple meningiomas (6%) are not shown.

We obtained advanced molecular profiling to supplement our histopathologic analysis of a prospective series of meningiomas with molecular data from May 2019 to April 2021. A total of 156 cases of meningioma were resected at OU Health during this two-year period and a minimal number of cases were excluded from this cohort (**Figure 1A**). Of the five cases that were excluded from analysis, two lacked molecular data due to insufficient DNA, one was located outside of the CNS, and two were duplicate cases that recurred within the two-year period of this study. The remaining 151 cases that were included in analysis comprised mostly WHO grade 1 cases (85%), but ~15% were WHO grade 2/3 (**Figure 1B** and **Supplementary Table 1**). Alterations in the *NF2* gene, the most common genetic disturbance associated with meningiomas, were evenly distributed across grades and comprised approximately 40% of the total meningioma cohort. The common histopathologic subtypes were all represented in our cohort with meningothelial and transitional comprising the majority of grade 1 tumors (**Figure 1C**) (1, 3). Demographics of our cohort also aligned with national data with grade 1 meningiomas occurring more frequently in females, while this discrepancy diminishes in WHO grades 2 and 3 (**Figure 1D** and **Supplementary Table 1**) (3, 6). Molecular data obtained from Mayo Clinic Laboratories included the Neuro-Oncology Expanded Gene Panel which reports sequence alterations in 118 genes and copy number alterations determined by chromosomal microarray. Sequence alterations were probed by the Neuro-Oncology Expanded Gene Panel for 99% of cases. Copy number alterations were determined by chromosomal microarray for 82% of cases. 81% of cases had data from both the Neuro-Oncology Expanded Gene Panel and chromosomal microarray (**Figure 1E** and **Supplementary Table 1**). The cohort included mostly intracranial meningiomas with only 2.6% of cases located in the spine and 6% of cases with multiple meningiomas. Meningiomas arising from the meninges overlying the anterior and posterior convexities, the falx, and tentorium were included in the cohort, and more than half of cases (52%) were classified as skull base meningiomas (**Figure 1F**).

### Copy Number and Sequence Alterations Consistent With High Grade Seen in a Significant Number of WHO Grade I Meningiomas

**Figure 2:**
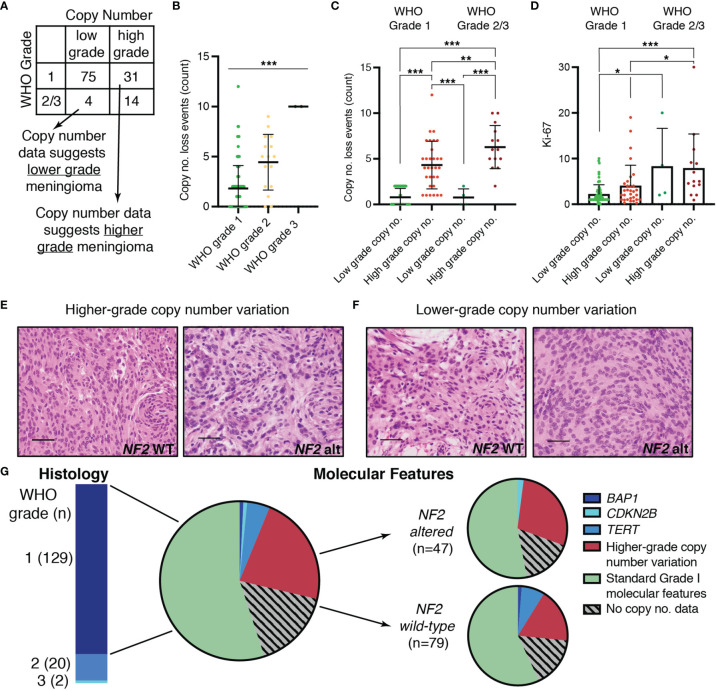
Cases with inconsistencies between molecular data and histopathologic WHO grading. **(A)** Comparison of “molecular grade” suggested by copy number data with histopathologic WHO grade. **(B)** Absolute count of chromosome arms with copy number loss events in any of: 1p, 3p, 4p/q, 6p/q, 10 p/q, 14q, 18p/q, 19p/q, 22 p/q in each WHO grade. **(C)** Absolute count of copy number loss events for cases with low-grade or high-grade copy number profiles in WHO grade 1 and WHO grade 2/3 meningiomas. **(D)** Scatter plot showing maximum Ki-67 for WHO grade 1 and WHO grade 2/3 meningiomas with low-grade or high-grade copy number profiles. **(E, F)** Representative images showing hematoxylin and eosin (H&E)-stained slides at 40X demonstrating the histomorphology of *NF2* wild-type (left) and *NF2* altered (right) meningiomas with **(E)** higher-grade or **(F)** lower-grade copy number profiles. **(G)** Pie chart depicting the proportion of WHO grade 1 cases with higher-grade copy number profiles or with sequence alterations in *BAP1, CDKN2B*, or *TERT* in the total cohort (middle) and in *NF2* wild-type versus *NF2* altered meningiomas (right). Mutations in the *TERT* promoter were seen 17% (1/6) of the *TERT* alterations. For panels **(B–D)**, mean and standard deviation is shown and statistical significance is indicated by asterisks (*p < 0.05, **p < 0.01, ***p < 0.001).

Copy number data have been shown to predict tumor behavior, and several groups have proposed molecular grading systems based on copy number data due to the accessibility of this data in a non-research clinical setting (6, 10, 12, 23, 24). We determined whether meningiomas in our cohort had copy number profiles consistent with low-grade or high-grade meningiomas – thereby classified as “low” or “high” risk profiles – based on prior literature. These profiles were then compared to the assigned histopathologic WHO grade (**Figure 2A**). Copy number events were frequently loss events and increased significantly with increasing WHO grade (p < 0.0001) (**Figure 2B**). Grade 1 meningiomas generally have more balanced copy number profiles or isolated monosomy 22. WHO grade 1 meningiomas with higher grade copy number profiles have been shown to progress to a higher WHO grade over time (6, 25). Aside from chromosome 22, loss of 1p was the most common chromosomal abnormality in WHO grade 1 as well as WHO grade 2/3 in our series, consistent with prior reports. Losses involving 6q and 14q also occurred frequently. (**Supplementary Table 2**). Low grade copy number profiles were seen in 71% of WHO grade 1 meningiomas of which 44% had no significant copy alterations. Surprisingly, the remaining 29% of WHO grade 1 meningiomas had copy number profiles suggestive of a higher-grade tumor and were therefore referred to as “higher risk” profiles. For WHO grade 2/3 meningiomas, 78% of cases had copy number profiles consistent with high grade, but 22% exhibited copy number profiles usually found in grade 1 meningiomas (**Figure 2A**). The difference in copy number loss events for meningiomas with low grade copy number profiles was not statistically different between WHO Grade 1 when compared to WHO Grade 2/3 meningiomas (p > 0.999). Copy number loss events were significantly higher for cases with higher-grade copy number profiles when compared to those with lower grade copy number profiles regardless of WHO grade (p < 0.001) (**Figure 2C**). The mitotic index (Ki-67) was higher for WHO grade 1 meningiomas with high grade copy number profiles when compared to WHO grade 1 meningiomas with low grade copy number profiles (4.0 ± 4.5 vs. 2.2 ± 2.08). However, there was considerable variability, and this difference did not reach statistical significance (p = 0.14) (**Figure 2D**). Similarly, the mitotic index was not statistically different between WHO grade 2/3 meningiomas with low risk compared to high-risk copy number profiles (p = 0.998). WHO grade 1 meningiomas with high risk molecular profiles were not distinguishable from those with low risk molecular profiles based on histologic architecture alone (**Figures 2E, F**). In addition to copy number alterations, sequence alterations in *BAP1*, *CDKN2A/B*, and *TERT* are associated with a more aggressive clinical course. In our cohort, 8% of meningiomas harbored mutations in one of these genes with most of these cases (8 out of 12) occurring in WHO grade 1 meningiomas (**Figure 2G**). These discrepancies occurred in both *NF2* wild-type as well as *NF2-*altered meningiomas, although higher grade copy number alterations were more common in *NF2*-altered meningiomas. *TERT* mutations occurred more frequently in *NF2* wild-type meningiomas. Mutations in the *TERT* promoter accounted for 29% of *TERT* mutations. Interestingly, most cases with sequence alterations in *BAP1*, *CDKN2A/B*, and *TERT* occurred in meningiomas with a low-grade cytogenetic background. Taken together, higher-risk copy number profiles or adverse mutational profiles were present in 29% of WHO grade 1 cases, and these were not distinguished by mitotic index nor histologic architecture.

### Genomic Landscape of the Cohort Identifies Molecular Subpopulations of Meningiomas

**Figure 3:**
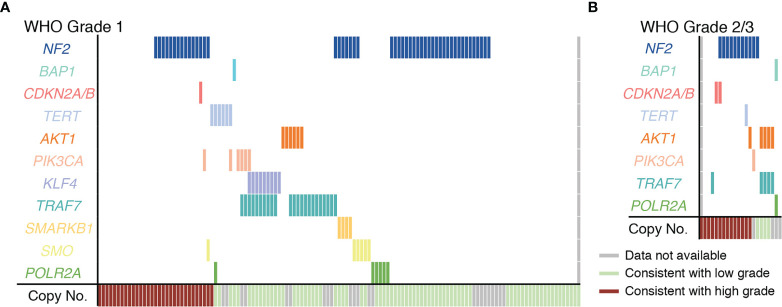
Genomic landscape of meningiomas included in the cohort. **(A)** Depiction of all Grade 1 meningioma cases harboring high grade (red) or low grade (green) copy number profiles and sequence alterations as determined by advanced molecular profiling. **(B)** Plot showing copy number alterations and sequence alteration for all WHO grade 2 and 3 cases included in the study. For both **(A, B)** each vertical column represents one case. *TERT* mutations were in the promoter in 17% (1 case) of WHO Grade 1 meningiomas **(A)** and in all of the WHO grade 2 *TERT* mutated meningiomas (1 case) **(B)**.

Efforts to better understand the biology of meningiomas have identified mutually exclusive subpopulations of WHO grade 1 meningiomas with unique driver mutations. These include meningiomas with mutations in *AKT1*, *PIK3CA*, *POLR2A*, *SMO*, *KLF4*, and POLR2A, *TRAF7*. Mutations in these genes have been shown to correlate with tumor location and, in some cases, determine histologic subtype of meningioma or inform tumor behavior (6, 16, 26, 27). A focused mutational profile of our cohort is shown in **Figure 3A**. Mutational profiles subdivided meningiomas into genetically distinct subgroups most of which had either a single oncogenic driver mutation or previously described co-mutations such as *KLF4* K409Q (K443Q) with an altered *TRAF7* or *AKT1* E17K with an altered *TRAF7* (**Figure 3A** and **Supplementary Table 3**). With few exceptions, mutations occurred in a bland cytogenetic background, which is consistent with these mutations being associated with a more benign clinical course when compared to meningiomas with high-grade copy number alterations (6, 24, 25). Since most meningioma cases in our cohort were WHO grade 1, the genomic data for our WHO grade 2/3 meningiomas is more limited (**Figure 3B**). Nevertheless, our analysis of WHO grade 2/3 meningiomas is consistent with previous reports of the genomic landscape seen in higher WHO grade meningiomas (25). While *NF2* alterations are frequently encountered in WHO grade 2/3 meningiomas, the abovementioned driver mutations encountered in *NF2* wildtype meningiomas occur less frequently in grade 2/3 meningiomas. One exception to this was a recurring co-mutation *AKT1* and *TRAF7* that occurred in higher grade meningiomas with a low-grade cytogenetic background.

### Correlation in Demographics and Tumor Location Are Seen in Meningiomas With Similar Molecular Profiles

**Figure 4:**
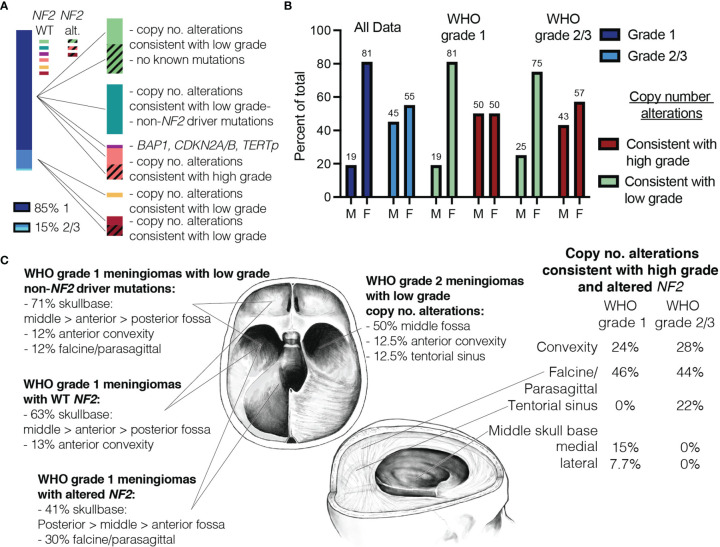
Similarities in demographics and tumor location are seen in meningiomas with similar copy number data. **(A)** Proportion of grade 1 vs. grade 2/3 meningiomas based on histopathologic grading shown on left. Meningiomas divided by molecular data (right). **(B)** Male-to-female ratio plotted side by side for all WHO grade 1 and grade 2/3 meningiomas (blue), meningiomas with copy number alterations consistent with low grade (green) and those consistent with high grade (red). **(C)** Meningiomas with low grade copy number alterations predominately occur in skull base locations (WHO grade 1 meningiomas shown on left). WHO grade 2 meningiomas with low grade copy no. alterations and meningiomas with high grade copy no. alterations are shown on right.

Molecular profiling allowed us to further subdivide meningiomas into several molecular sub-groups that are predicted to behave similarly based on previous studies (**Figure 4A**). Of note, sub-groups with low-grade copy number and sequence alterations exhibited similar ratios of M:F (~25:75) regardless of WHO grade (**Figure 4B**). Furthermore, meningiomas with low grade features tended to occur more frequently in skull base locations with *NF2* wild-type meningiomas occurring more frequently in the anterior and middle fossae, whereas *NF2-*altered meningiomas were more commonly located in the posterior and middle fossae and along the falx (**Figure 4C**). In contrast, meningiomas with higher grade copy number alterations had more balanced M:F ratios which is often seen in higher grade meningiomas (**Figure 4B**). Furthermore, these tended to occur more frequently in paravenous locations (i.e., parasagittal, falcine) and rarely occurred in the anterior skull base where grade 1 meningiomas more frequently occurred (**Figure 4C**) (25).

## Discussion

Molecular data have been incorporated in the WHO grading criteria for other CNS tumors since 2016, while grading of meningiomas has been largely based on histopathologic data (5). This year, molecular alterations, including *CDKN2A/B* and *TERTp*, were added to the WHO classification for meningiomas (21). While alterations in these genes are recognized as negative prognostic markers, they occur in a small number of meningiomas and do not aid in identification of intermediate risk meningiomas (7). In recent years, there has also been a rapidly growing body of literature supporting the use of advanced molecular profiling in classifying meningiomas. Classification schemes based on methylation, sequence alterations, and copy number data have been introduced and have been shown to be superior to WHO grading in predicting tumour behaviour (10, 12–14). Nevertheless, such molecular methods are not widely incorporated in clinical practice where histopathologic WHO grade remains the standard that guides management of patients with meningioma.

A major hurdle to incorporation of advanced molecular profiling in the routine care of patients with meningiomas is that access to advanced molecular profiling methods, including DNA and RNA sequencing as well as methylome studies, is limited to major academic and research institutions (28, 29). To address this, we used resources that are commercially available for routine determination of tumour genetics through the Mayo Clinic Laboratories and assessed the clinical value of incorporating advanced molecular profiling of all resected meningiomas into routine clinical management. The advanced molecular profiling methods employed in this study are ones that could therefore feasibly be accessed widely by treatment groups in the management of meningiomas. While this additional testing adds upfront cost to the evaluation of meningiomas, the potential benefits of accurately predicting clinical behaviour and improving clinical management are invaluable, potentially avoiding unnecessary treatment in patients with WHO grade 2/3 tumors expected to be low-risk and possibly improving outcomes for WHO grade 1 meningiomas with high-risk genetic profiles.

An important consideration in the management of meningiomas is the use of adjunct radiation for treatment of tumors that are difficult to control with surgical resection alone. Recent trials have evaluated the benefits of radiation in meningiomas with an intermediate risk of recurrence, including recurrent grade 1 meningiomas and grade 2 meningiomas following gross-total resection (8, 9), while randomized trials are ongoing. While the proper use of radiation in this subset of meningiomas remains an area of debate, proper and consistent classification of meningiomas into low-, intermediate-, and high-risk categories is crucial in order to accurately evaluate the appropriateness of radiation therapy in management (7, 30). Systemic therapies targeting oncogenic driver mutations have had limited success in the treatment of meningiomas and are not widely incorporated in clinical practice (31, 32). However, molecular targets continue to be an area of research for meningiomas with ongoing clinical trials (33). If systemic therapies become a viable option for treatment of meningioma, molecular data will be crucial for clinical decision making.

We found a significant percentage of meningiomas within our cohort that were predicted to behave inconsistent with their assigned WHO grade based on the molecular profiles (nearly 30%). This is consistent with a recent report that applied advanced genomics to create a molecular grade with improved prediction of meningioma behaviour, with up to 32% of cases reclassified when molecular data were applied (10). There are limitations inherent to our study design that are important to consider. While our sample generally mirrors national data, we include a slightly higher proportion of grade 1 cases (85% vs. the reported 80% (2). With a sample size of 151 patients, we have a cohort with relatively small numbers of grade 2 cases and only two grade 3 cases. Our cohort is also fairly heterogenous, including cranial and spinal cases as well as eleven recurrent cases, and with a larger fraction of skull base tumors. While such heterogeneity can introduce variables that confound interpretation, we felt that for the purpose of this study it was important to minimize the exclusion of cases to accurately represent a two-year meningioma cohort. The data we have included are from recently resected meningiomas; therefore, we do not include prospective data providing patient follow-up and critical metrics, such as rates of recurrence of meningiomas and overall survival. The goal of this study was to quantify the rate of discrepancy between molecular profiles and histopathologic grading; however, patient follow-up to determine the predictive power of the molecular profile when compared to histopathologic grade will be a critical next step in reconciling molecular data with histopathologic grade.

In recent years, the clinical impact of molecular data in the treatment and management of meningiomas has become evident. Numerous classifications that incorporate molecular data have been shown to improve the prediction of tumour behaviour for meningiomas. With the rapid advances in molecular understanding of meningiomas over the recent years, it will be important to determine how this information can be integrated in routine clinical settings and standardized nationally, in particular for institutions with limited resources, and to correlate this information with clinical outcomes.

## Data Availability Statement

The original contributions presented in the study are included in the article/**Supplementary Material**. Further inquiries can be directed to the corresponding author.

## Ethics Statement

The studies involving human participants were reviewed and approved by Institutional Review Board University of Oklahoma Health Sciences Center. Written informed consent for participation was not required for this study in accordance with the national legislation and the institutional requirements.

## Author Contributions

Study design and data analysis: AR, PP, and ID. Histopathologic grading, processing, and shipment of meningioma samples for advanced molecular profiling: JP and K-MF. Drafting of manuscript: AR and PP. Proofreading, finalizing, and approval of manuscript: all authors.

## Conflict of Interest

The authors declare that the research was conducted in the absence of any commercial or financial relationships that could be construed as a potential conflict of interest.

## Publisher’s Note

All claims expressed in this article are solely those of the authors and do not necessarily represent those of their affiliated organizations, or those of the publisher, the editors and the reviewers. Any product that may be evaluated in this article, or claim that may be made by its manufacturer, is not guaranteed or endorsed by the publisher.
