# Supplementary material for: The Discrepancy Between Standard Histologic WHO Grading of Meningioma and Molecular Profile: A Single Institution Series
Source: Front Oncol. 2022 Mar 1;12:846232. doi: 10.3389/fonc.2022.846232 (PMC8921552; doi:10.3389/fonc.2022.846232)
Supplement: Supplementary file 1 [file DataSheet_1.pdf]

Supplementary Material

1 Supplementary Figures

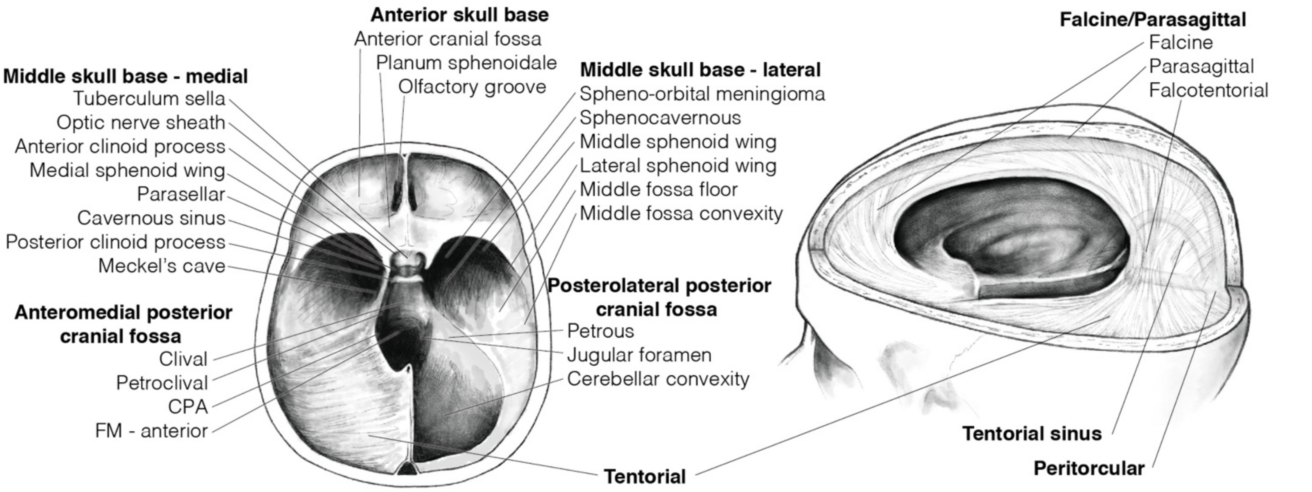

Supplementary Figure 1. Superior view of skull base meninges (left) and superolateral view of meninges (right) showing the classification and locations of meningiomas. Locations not depicted in this illustration are anterior convexity meningiomas (convexity anterior to central sulcus, frontotemporal convexity), posterior convexity meningiomas (convexity posterior to central sulcus), intraventricular, and spinal meningiomas. FM = foramen magnum.

2 Supplementary Tables

Supplementary Table 1: OU Health meningioma cohort

		**All meningiomas**	**WHO Grade 1**	**WHO Grade 2**	**WHO Grade 3**
n (%)		151 (100)	129 (85.4)	20 (13.3)	2 (1.3)
Male n (%)		35 (23)	25 (20)	9 (45)	1 (50)
Female n (%)		116 (77)	104 (80)	11 (55)	1 (50)
Age mean (range)		59 (22-88)	59 (22-88)	59 (25-74)	74 (67-81)
**Histology**					
I	Meningothelial	74 (49)	74 (57)		
	Transitional	34 (23)	34 (26)		
	Psammomatous	4 (2.6)	4 (3.1)		
	Angiomatous	4 (2.6)	4 (3.1)		
	Secretory	3 (2.0)	3 (2.3)		
	Fibroblastic	3 (2.0)	3 (2.3)		
	Microcystic	1 (0.7)	1 (0.8)		
	Lymphoplasmacyte-rich	0 (0.0)	0 (0.0)		
	Metaplastic	0 (0.0)	0 (0.0)		
	>1 histologic subtype	5* (3.3)	5* (3.9)		
	Undetermined	1 (0.7)	1 (0.8)		
II	Atypical	17 (11)		17 (85)	
	Chordoid	3 (2.0)		3 (15)	
	Clear-cell	0 (0.0)		0 (0.0)	
III	Papillary	1 (0.7)			1 (50)
	Rhabdoid	0 (0.0)			0 (0.0)
	Anaplastic	1 (0.7)			1 (50)
Max Ki-67/MIB-1 Mean (range)		3.7 (0.06-32.0)	2.7 (0.06-19)	8.4 (0.9-32)	19.5 (8.9-30)
***NF* status**					
NF altered n (%)		60 (40)	49 (38)	10 (50)	1 (50)
NF wildtype n (%)		91 (60)	80 (62)	10 (50)	1 (50)
**Molecular Profiles included**					
Cytogenetic data		124	106	16	2
Neuro-oncology panel		149	128	19	2

*These specimens equally exhibited two histologic subtypes. The majority of these were a combination of meningothelial and transitional, pasmmomatous, or secretory histology.

Supplementary Table 2: OU Health meningioma cohort

Copy number locus	Overall prevalence (n = 124)	Grade 1 – low grade copy no. (n = 75)	Grade 1 – high grade copy no. (n = 31)	Grade 2 – low grade copy no. (n = 4)	Grade 2 – high grade copy no (n = 12)	Grade 3 – high grade copy no (n = 2)
1p	39 (31%)	1 (1.3%)	24 (77%)	0 (0%)	12 (100%)	2 (100%)
3p	10 (8.1%)	0 (0%)	6 (19%)	0 (0%)	3 (25%)	1 (50%)
4p	9 (7.3%)	0 (0%)	6 (19%)	0 (0%)	2 (17%)	1 (50%)
4q	5 (4.0%)	0 (0%)	1 (3.2%)	0 (0%)	3 (25%)	1 (50%)
6p	10 (8.1%)	0 (0%)	6 (19%)	0 (0%)	4 (33%)	0 (0%)
6q	16 (13%)	0 (0%)	8 (26%)	1 (25%)	7 (58%)	0 (0%)
10p	9 (7.3%)	0 (0%)	4 (13%)	0 (0%)	3 (25%)	2 (100%)
10q	12 (9.7%)	0 (0%)	6 (19%)	0 (0%)	4 (33%)	2 (100%)
14q	21 (17%)	0 (0%)	12 (39%)	0 (0%)	8 (67%)	1 (50%)
18p	8 (6.5%)	0 (0%)	5 (16%)	0 (0%)	2 (17%)	1 (50%)
18q	13 (11%)	0 (0%)	8 (26%)	0 (0%)	3 (25%)	2 (100%)
19p	11 (8.9%)	0 (0%)	8 (26%)	0 (0%)	1 (8.3%)	2 (100%)
19q	5 (4.0%)	0 (0%)	4 (13%)	0 (0%)	0 (0%)	1 (0%)
22p	53 (43%)	28 (38%)	15 (48%)	1 (25%)	7 (58%)	2 (100%)
22q	67 (54%)	31 (42%)	22 (71%)	1 (25%)	11 (92%)	2 (100%)
None	35 (28%)	33 (44%)	0 (0%)	2 (50%)	0 (0%)	0 (0%)
Other gain/loss	59 (48%)	20 (27%)	25 (81%)	0 (0%)	12 (100%)	2 (100%)

Supplementary Table 3: Molecular profile of meningiomas with identified driver mutations

Gene	Mutation	Cytogenetic Background	WHO Grade	Co-mutated Genes			
				Gene	Mutation	Gene	Mutation
AKT1	E17K	Low grade	1	None	-		
	E17K	Low grade	1	None	-		
	E17K	Low grade	1	*TRAF7*	R641H		
	E17K	Low grade	1	*TRAF7*	G536S		
	E17K	Low grade	1	*TRAF7*	K615E		
	E17K	Low grade	1	*TRAF7*	N520S		
	G109S	Low grade	2	*NF2*	W184*		
	E17K	Low grade	2	*TRAF7*	N520S		
	E17K	Low grade	2	*TRAF7*	P398R		
	E17K	Low grade	2	*TRAF7*	R653P		
	E17K	Low grade	2	*TRAF7*	K615E		
PIK3CA	H1047L	Low grade	1	None	-		
	V71I	**High grade**	1	*NF2*	241-1G>C		
	H1047R	Low grade	1	*TERT-alt*	E441del		
	E545A	Low grade	1	*TRAF7*	N520S		
	N497S	Low grade	1	*TRAF7*	N520S	*KLF4*	K409Q (K443Q)
	C420R	Low grade	1	*TRAF7*	C388Y		
	E81D	Low grade	2	*NF2*	R172Kfs*31		
BAP1	E491*	Low grade	1	None	-		
	G185R	Low grade	2	*POLR2A*	S1562T		
CDKN2A/B	A88T	**High grade**	1	*NF2*	241-1_241delinsCT		
	R101Q	**High grade**	2	None	-		
	E26Gfs*24	**High grade**	2	*NF2*	F96del		
KLF4	A122T	Low grade	1	None	-		
	K409Q (K443Q)	Low grade	1	*TRAF7*	N520S		
	K409Q (K443Q)	Low grade	1	*TRAF7*	N520S	*PIK3CA*	N497S
	K409Q (K443Q)	Low grade	1	*TRAF7*	K615E		
	K409Q (K443Q)	Low grade	1	*TRAF7*	G536S		
	K409Q (K443Q)	Low grade	1	*TRAF7*	G536S		
	K409Q (K443Q)	Low grade	1	*TRAF7*	G559R		
	K409Q (K443Q)	Low grade	1	*TRAF7*	R653Q		
	K409Q (K443Q)	Low grade	1	*TRAF7*	T391I		
POLR2A	P1746L	Low grade	1	None	-		
	I399K	Low grade	1	None	-		
	Q403K	Low grade	1	None	-		
	Q403K	Low grade	1	None	-		
	Q403K	Low grade	1	None	-		
	Q403K	Low grade	1	*TERT-alt*	E441del		

	S1562T	Low grade	2	*BAP1*	G185R		
SMARCB1	L356*	Low grade	1	*NF2*	R341*		
	L356Dfs*4	Low grade	1	*NF2*	E463Rfs*32		
	R377H/R386H	Low grade	1	*NF2*	E348*		
	R377H/R386H	Low grade	1	*NF2*	448-15_448-2del		
SMO	L412F	Low grade	1	None	-		
	L412F	Low grade	1	None	-		
	R772C	Low grade	1	None	-		
	V54M	Low grade	1	*NF2*	1122+1_1122+22del		
	P698R	Low grade	1	*NF2*	A388Pfs*38		
	P116S	**High grade**	1	*NF2*	L241Ffs*10		
TERTp[1]	.-124C>T	**High grade**	1	None	-		
	.-124C>T	**High grade**	2	*NF2*	L46R		
TERT-alt[1]	R972C	Low grade	1	None	-		
	E441del	Low grade	1	None	-		
	E441del	Low grade	1	None	-		
	E441del	Low grade	1	*PIK3CA*	H1047R		
	E441del	Low grade	1	*POLR2A*	Q403K		
TRAF7	Q643_G644delinsR	Low grade	1	None	-		
	T391A	Low grade	1	None	-		
	F354_R356del	Low grade	1	None	-		
	N520S	Low grade	1	None	-		
	G390E	Low grade	1	None	-		
	C431F	Low grade	1	None	-		
	G390R	Low grade	1	None	-		
	R653Q	Low grade	1	None	-		
	R641H	Low grade	1	*Akt1*	E17K		
	G536S	Low grade	1	*Akt1*	E17K		
	K615E	Low grade	1	*Akt1*	E17K		
	N520S	Low grade	1	*Akt1*	E17K		
	N520S	Low grade	1	*KLF4*	K409Q (K443Q)		
	K615E	Low grade	1	*KLF4*	K409Q (K443Q)		
	G536S	Low grade	1	*KLF4*	K409Q (K443Q)		
	R653Q	Low grade	1	*KLF4*	K409Q (K443Q)		
	T391I	Low grade	1	*KLF4*	K409Q (K443Q)		
	G536S	Low grade	1	*KLF4*	K409Q (K443Q)		
	G559R	Low grade	1	*KLF4*	K409Q (K443Q)		
	N520S	Low grade	1	*KLF4*	K409Q (K443Q)	*PIK3CA*	N497S
	G437S	Low grade	1	*NF2*	W191*		

	C388Y	Low grade	1	*PIK3CA*	C420R		
	N520S	Low grade	1	*PIK3CA*	E545A		
	R641H	**High grade**	2	None	-		
	N520S	Low grade	2	*Akt1*	E17K		
	P398R	Low grade	2	*Akt1*	E17K		
	R653P	Low grade	2	*Akt1*	E17K		
	K615E	Low grade	2	*Akt1*	E17K		

[1]*TERTp* denotes alterations within the *TERT* promoter, while *TERT-alt* refers to mutations not within the promoter region.
